# MINE: maximally informative next experiment—toward a new GWAS experimental design and methodology

**DOI:** 10.1093/g3journal/jkaf163

**Published:** 2025-07-18

**Authors:** Isaac Torres, Shufan Zhang, Amanda Bouffier, Bernd Schüttler, Jonathan Arnold

**Affiliations:** Institute of Bioinformatics, University of Georgia, Athens, GA 30602, United States; Institute of Bioinformatics, University of Georgia, Athens, GA 30602, United States; Institute of Bioinformatics, University of Georgia, Athens, GA 30602, United States; Institute of Bioinformatics, University of Georgia, Athens, GA 30602, United States; Institute of Bioinformatics, University of Georgia, Athens, GA 30602, United States

**Keywords:** bioinformatics, GWAS, MCMC, experimental design

## Abstract

The computational methodology of Genome Wide Association Studies (GWAS) currently has several limitations: (i) the number of observations (rows) on a quantitative trait tends to be smaller than the number of single nucleotide polymorphisms (SNPs) (columns) in the design matrix; (ii) each SNP is usually modeled separately, failing to acknowledge interaction between each other (ie epistasis); (iii) there is implicit linkage disequilibrium (LD) between neighboring SNPs due to their linkage. To overcome these issues, we developed a tool that uses ensemble methods to fit mixed linear models to GWAS data, and these ensemble methods include the development of a new experimental design approach in GWAS, which uses the resultant models and data to select the next informative experiment over time. This new adaptive and staged approach for GWAS experimental design was developed and tested in a 3 yr adaptive model-guided discovery experiment against a fixed classical design. In *Sorghum bicolor* a total of 79, 86, and 78 accessions were tested in years 1, 2, and 3, respectively out of 343 accessions available in the Bioenergy Association Panel (BAP) each identified for 232,303 SNPs, 1 every 2–3 kb in the genomes. We demonstrated the feasibility of MINE enacted with 8 people in the field per year over 3 yr vs in 1 large classical design enacted with 20 people in 1 yr. The MINE results for chromosomal regions identified controlling dry weight were confirmed against results from previous sorghum GWAS experiments and 1 large classical design for the BAP panel.

## Teaser

In this work, Torres et al. have developed an experimental design tool for GWAS studies, as well as implemented new methodology on complex traits of sorghum. The experiment took place during 3 years on Wellbrook Farm, Watkinsville, GA at the University of Georgia.

## Introduction

Genome wide association studies (GWAS) have become a standard approach to gain insights about genes that control a complex trait, such as height (Yengo et al. 2022). As far as we know, there is no literature addressing GWAS experimental design (genotype selection) adaptively; in this work, we propose a method to select the annual most informative genotypes in a sequence of GWAS experiments for discovering chromosomal regions related to a quantitative trait. The advantage of this approach is to decompose a GWAS into a smaller series of more tractable field experiments that may be more informative than 1 large classical design. This approach is called MINE: maximally informative next experiment, and maximizes the prediction uncertainty volume in a complex trait from linear and mixed linear models; it was originally presented for analysis of *Neurospora crassa* genetic networks (Dong et al. 2008; McGee and Buzzard 2018; Torres et al. 2025), and it represents an alternative to classic experimental design (Fisher 1935, 1951; Ryan and Morgan 2007).

The modeling part of GWAS also allows us to rethink classic methodology in experimental design. In fact, linear regression is the technique used to fit linear models (Uffelmann et al. 2021) to GWAS data, where researchers generally have fewer individuals (rows) than single nucleotide polymorphisms (SNPs) or chromosomal regions (columns) in the design matrix. The solution proposed here for this problem is ensemble methods for fitting (Gelfand and Smith 1990). Geneticists regularly take 1 SNP at a time to create a model (1 model per SNP); therefore, a single model encapsulating available SNP interaction is not possible, and linkage disequilibrium (LD) is present implicitly due to the low number of bases separating SNPs. To overcome this problem a tool was created that puts together available SNPs forming wider chromosomal regions so that distinct regions are free of LD. The result was 1 matrix accounting for all SNPs, and in order to identify a model ensemble, methods are used and computed by Markov Chain Monte Carlo (MCMC). Specifically, the Metropolis algorithm (Metropolis et al. 1953) was used to compute the fit of both linear models with fixed effects and mixed linear models to data on complex traits from a GWAS. When we refer to linear models herein, it will be understood it is linear models with fixed effects throughout. To select the most significant chromosomal regions the Bayesian interval (Jaynes and Kempthorne 1976) method and Benjamini–Hochberg criteria (Benjamini and Hochberg 1995) were used in feature selection. A tool was also developed to extract the currently known genes within these chromosomal regions from the Phytozome database (Goodstein et al. 2012). Finally, the last issue is the choice of rows (ie accessions) to include in the design matrix for succeeding years of a GWAS. This design question was addressed by a particular MCMC method called MINE (Dong et al. 2008; McGee and Buzzard 2018; Torres et al. 2025).

In order to illustrate this new design methodology for GWAS, an adaptive GWAS was implemented on a bioenergy association panel (BAP) (Brenton et al. 2016) for *Sorghum bicolor* on Wellbrook Farm, Watkinsville, GA, over 3 yr. Roughly, 80 accessions each year were arranged in a randomized complete block design with 3 blocks over 3 yr to examine a variety of quantitative traits measuring plant health: log dry weight, tiller number, fungal disease burden, and height. There are over 10,000 genotypes worldwide that have been characterized in this tractable diploid genetic system for potential use in a GWAS (Hu et al. 2019).

## Materials and methods

### Field experimental design

The location for the field experiment was Wellbrook Farm in Watkinsville, Georgia, USA; ∼81 distinct accessions were planted each year for 3 yr randomly in 3 blocks. The list of accessions planted each year was overlapping because of the use of MINE described below. Each accession appeared 4–9 times in a plot. Plot sizes were 9, 6, and 4 in years 1, 2, and 3, respectively. Each plant was separated by 18 inches (in) in a plot. Each row was separated by 36 in. Each block was separated by 72 in. Accessions were randomized within a block by plot (ie row). The blocks were assessed to be uniform in soil composition by krigging measurements of soil samples.

The accessions were taken from the bioenergy accession panel (BAP) (Brenton et al. 2016), which means these accessions have already been sequenced to determine their SNP genotype (see next section); this BAP collected is maintained by the USDA in Griffin, GA.

### SNP genotyping by sequencing

Reduced representation sequencing libraries were constructed using ApekI and sequenced in an Illumina HiSeq 2000 (Brenton et al. 2016). Reads were processed in TASSEL 5.0 (Bradbury et al. 2007). Alignment was performed to the sorghum reference genome version 2 (Goodstein et al. 2012) by Burrows–Wheeler alignment (BWA) (Li and Durbin 2009). After trimming and filtering reads the result was 343 accessions and 232,303 SNPs for a SNP density of 2–3 kb in each BAP accession.

FASTQ files from the BAP data set (Brenton et al. 2016) were then merged with other Sorghum data sets (Hu et al. 2019), who then processed the reads with TASSEL 5.0, aligned them with BWA to the sorghum reference genome version 3.1 (Goodstein et al. 2012), identified SNPs with *DiscoverySNPCallerPluginV2* in TASSEL 5.0 (Bradbury et al. 2007), and carried out imputation with Beagle (Browning and Browning 2016). The resulting BAP data set was downloaded as a variant call format (VCF) file for use here in our study (Hu et al. 2019).

Initially the BAP seed were ordered through USDA and germinated in pots at a UGA greenhouse; watering was done daily, and after a period of 2 wk small sorghum seedlings were transplanted to Wellbrook Farm. Once in the soil at the farm, a weeding regime was established twice per week, and harvesting took place 3 mo later. During harvesting disease and height were determined and recorded; canopies were chopped and put into bags to be taken to ovens for drying and weighing. Foliar fungal disease (and referred to as disease hereafter) was scored for Leaf Blight, Target Leaf Spot, Zonate Leaf Spot, Gray Leaf Spot, and Anthracnose. Disease and height data were put directly in the database; however, dry weight canopies spent 1 wk in the oven and were weighed immediately thereafter. The dry weight was recorded in grams, height in meters, and disease with a number from 1 to 10 representing its severity on the leaves of the plant. All software developed and used in this work can be found at the following link: https://github.com/JArnoldLab/MINE. The VCF files from (Hu et al. 2019) are available at: https://doi.org/10.6084/m9.figshare.28548572.v1

### Modeling

GWAS modeling has traditionally used linear regression models (Uffelmann et al. 2021). The challenge with this approach is that the number of parameters *p* greatly exceeds the number of individuals *n* in practice. This is an old problem in statistical physics (Landau and Binder 2021), in which the goal was to model the motion of large number of particles in an ideal gas with only 3 measurements, pressure, volume, and temperature (Guggenheim 1955). Instead of finding 1 solution, Boltzmann opted to find an ensemble Q(β,X) of solutions with *β* being the parameters and X being the design, consistent with the available data, to make predictions about the system. (A glossary of symbols can be found in the Appendix). This ensemble as used here is a probability distribution on the parameter space, and model averaging is used to predict the behavior of the system. More recently, ensemble methods were first introduced into systems biology to describe genetic network behavior (Battogtokh et al. 2002). Any ensemble method begins with the ensemble Q(β,X), and in this GWAS context is a probability distribution over a collection of linear models. The ensemble Q(β,X) is usually associated with a Hamiltonian *H* and specifies Q(β,X)∝exp(−H) to obtain the Boltzmann factor for a particular system.

In order to make predictions about any system including in a GWAS, the ensemble Q(β,X) needs to be identified according to some fitting criterion, such as the size of the ensemble probability Q(β,X). MCMC methods are used to optimize this fitting criterion (Battogtokh et al. 2002; Landau et al. 2014; Landau and Binder 2021). These MCMC methods perform a random walk in the parameter space in order to make the probability Q(β,X) large or equivalently, the Hamiltonian H small. MCMC methods generally have 2 stages in this search for a collection of models identifying the ensemble. The first stage is referred to as an equilibration stage or burn-in (Battogtokh et al. 2002; Landau and Binder 2021), in which promising regions of the parameter space are sought. The second stage is referred to as an accumulation phase, in which models are accumulated in the promising region to specify the ensemble. The collection of models obtained in the accumulation phase is used to make predictions about the system. Both the equilibration and accumulation phases are indexed by the sweep. A sweep is by definition, a visit to all parameters in the model on average once during a portion of the random walk. If there are 2,748 parameters in a GWAS, then a sweep will consist of 2,748 iterations of the MCMC experiment. A sweep is introduced as a way to produce a collection of parameters in the ensemble that are approximately uncorrelated. In fact to increase the likelihood of this decorrelation, sometimes sweeps are used to separate selected models for the accumulation phase and are not incorporated into the model ensemble. Here, for example, 1,000 decorrelation sweeps were typically used to separate models entering the ensemble in the accumulation phase. Each MCMC experiment is typically 1,000,000 sweeps in size.

MCMC methods for implementing ensemble methods are a good alternative when the number of observations is less than the number of parameters (Gelfand and Smith 1990), and the Metropolis algorithm was used as a backbone for solving the optimization of the ensemble Q(β,X) with respect to the parameters $\beta =(\beta_{1},\ldots ,\beta_{p}{)}^{\prime }$ as a random walk in the parameter space. The transpose (′) implies *β* is a column vector. The random walk is generated by a uniform random number generator *U*(−1,1) on the interval [−1,1]:

**Algorithm 1.** (Customized Metropolis Algorithm)

*Set a stepwidth*
*Set N_e_ number of equilibration sweeps*
*Set p number of parameters in the β vector*
*Set N_d_ number of decorrelation sweeps*
*Set M number of β vectors to accumulate*
*Set a random initial β vector*
*for N_e_ times do*
*for p times do*
*Choose an element β_k_ from β randomly*
*Propose an update β_k_*′ *= β_k_ + (stepwidth ∗ U(−1, 1))*
*Accept the change with probability min(1, Q(β′, X)/Q(β, X))*
*for M times do*
*for N_d_ times do*
*for p times do*
*Choose an element β_k_ from β randomly*
*Propose an update β_k_ = β_k_ + (stepwidth ∗ U(−1, 1))*
*Accept the change with probability min(1, Q(β′, X)/Q(β, X))*
*Store the β vector*
***return*** *Accumulate β vectors*

This Metropolis Algorithm 1. is an implementation of a random walk in the parameter space of all βs. Whenever a step is proposed in the random walk, which decreases the Hamiltonian, the algorithm will take this step. The key feature of the algorithm is that it will sometimes move uphill if the next step leads to a value of the Hamiltonian not too much higher than the current value. In the algorithm, a biased coin is tossed to decide on whether or not to take an uphill move. A proposed move on the Hamiltonian surface is less likely when it moves to a higher value. In this way, the Metropolis algorithm avoids being trapped in a local minimum of the Hamiltonian. The Metropolis algorithm with its uphill moves gives a means to exit from local minima on the Hamiltonian surface. Execution of this algorithm usually involves *N_e_* = 8,000–10,000, *p* = 2748, *P* = 1000, and *M* = 1000.

In order to use all of the data in the fitting procedure, a design matrix was introduced (Regular GWAS methodology considers 1 SNP at a time, and consequently no SNP interactions and carries LD implicitly). A tool was implemented that puts all available SNPs together to form chromosomal regions, a method known as the sum method (Romagnoni et al. 2019). The decay of LD occurs over a range of 3.5–35.5 kb in *S. bicolor* (Wang et al. 2013). The size of each chromosomal region was increased by adding adjacent SNP(s) until the size of the region was at least 50 kilobases (kb). Thus, SNPs from different chromosomal regions are unlikely to be in LD due to the mating system. To generate the elements of the design matrix X*_ij_* for the *i*th accession in the *j*th chromosomal region, an allelic count of the reference allele within the region was made and normalized. This yielded a design matrix X of 2,748 columns covering the entire genome and sidestepped the issue of LD due to linkage (Wang et al. 2022). Lewontin and Kojima (1960), however, demonstrated that there are at least 2 sources of LD in complex polymorphisms, linkage and epistasis. The latter still remains to be detected between markers, thus the need to consider simultaneously all markers rather than 1 at a time.

SNPs from different chromosomal regions are unlikely to be in LD due to historical recombination or lack thereof; this yields a design matrix that covers the entire genome if desired and sidesteps the issue of LD due to linkage (Weir 1990). The tool is designed to work individually with each chromosome, so if the user wanted to cover all of the genome, an additional tool was implemented to join the smaller matrices coming from each chromosome into 1 large design matrix.

### Linear model

The Metropolis algorithm is based on a particular model for GWAS, which is then used to setup a minimization problem characterizing model fit; the first model chosen was a linear model with fixed effects using the previously defined design matrix. The model is defined as follows to predict the measurements of the complex trait listed in the vector components of $Y=(Y_{1},\ldots ,Y_{n}{)}^{\prime }$:


(1) Y=Xβ+ε

The Greek letter *β* represents the vector of parameters describing the effects of particular chromosomal regions (design matrix columns) formed from SNPs. In total there were 2,748 regions in the *S. bicolor* genome. The rows of the X matrix (design matrix) represent the genotypes of each accession. A component of the vector *ɛ* is the error in 1 observation on 1 accession, and the vector components of ϵ are independently and identically distributed with variance $\sigma^{2}.$ The criterion for identifying the ensemble is that of minimizing the Hamiltonian or equivalently maximizing the ensemble probability Q(β,X). For the model in Equation (2), the Hamiltonian is taken as:


(2) $$H(\beta ,X)=\;(Y-X\beta {)}^{\prime }V^{-1}(Y-X\beta )$$

This formula represents a distance metric of how far the model prediction is from the observed value of the quantitative trait and is usually referred to as the sum of squared errors. The *V* matrix is block diagonal and fixed at the sample variances in the complex trait for each accession. The Metropolis algorithm carries out a random walk in the parameter space to minimize the Hamiltonian. In order to determine if the proposed random step is good or not, the Metropolis algorithm utilizes a probability function. The Boltzmann factor was chosen and is very well-known in statistical physics (Landau and Binder 2021). Its definition is the following:


(3) $$Q(\beta ,X)=\;\frac{1}{\Omega (X)}\;e^{-H(\beta ,X)}$$

The normalization constant Ω(X) is obtained by integrating over the parameter space of all *β*.

The Metropolis algorithm implementation has 2 phases; the first phase is to equilibrate the system at a local minimum in the Hamiltonian, which means that toward the end of the equilibration stage the parameters being estimated change very little. We say that they reached an equilibrium in the MC experiment. The second accumulation phase comprises collecting the M almost best solutions. In our case 1,000 sets of parameters were collected to characterize the ensemble.

### Mixed linear model

The mixed linear model is an elaboration of the linear model and a standard now for GWAS (Zhang et al. 2010). We included variance components into the effects of different chromosomal regions, and it is defined as follows:


(4) Y=Xβ+Zu+ε

The first term (*Xβ)* represents the trait prediction, such as biomass; the second term (*Zu*) represents the variance components and heritability, and it is defined as:


(5) $$V=\;\overset{n}{\underset{i=1}{\sum }}\;Z_{\;ji)}Z_{\;j(i)}^{\prime }\sigma_{\;j(i)}^{2}+\;\sigma^{2}I$$

The index *i* runs over n plant samples. Some samples being the same accession share the same variance component. The index *j*(*i*) returns the variance component of the *i*th observation for the *j*th accession. In Equation (5) throughout the paper, we take $Z_{i}=X_{i}.\;\;\mathrm{The}\;\mathrm{Hamiltonian}\;\mathrm{for}\;\mathrm{the}\;\mathrm{mixed}\;\mathrm{linear}\;\mathrm{model}\;$ can be obtained from the negative natural log of the ensemble (*H* = −ln*Q*):


(6) $$Q(\beta ,X)=\;\frac{e^{-.5{\left(Y-X\beta \right)}^{\prime }V^{-1}(Y-X\beta )}}{{\left(2\pi \right)}^{n/2}{\left|V\right|}^{1/2}}$$

(7) $$H(\beta ,X)=\;\frac{1}{2}(Y-X\beta {)}^{\prime }V^{-1}(Y-X\beta )+\;\frac{1}{2}nln(2\pi )+\frac{1}{2}\ln(\left|V\right|)$$

The Boltzmann factor is defined as proportional to exp(−H) and was chosen to be (7) so that the resulting distribution Q(β,X) was multivariate normal for the mixed linear model (Landau and Binder 2021). The resulting Hamiltonian has 2 more terms than the linear model with fixed effects. One of the additional terms contains the transcendental number π=3.1457…, and | | is a shorthand for the determinant of a matrix.

### Stepwidth adjuster feature in the metropolis algorithm

In the Metropolis algorithm model, parameters are sampled in random steps from the current position in the parameter space around a region defined by the local minimum in the Hamiltonian; however, the random step depends on a number selected by the user, the stepwidth; later, the random step is accepted or rejected. It is recommended that the rate of accepted steps is between 30% and 70% during the equilibration phase; this acceptance rate range is not always achieved, and may be an effect of being stuck in a small subregion during the MC experiment. Consequently, the parameters collected in the accumulation phase (MC sample) for fixed step width for each parameter may include models that represent a bad fit mixed in with the almost best solutions.

To overcome this issue, a dynamic stepwidth adjuster for stepwidth *S_i_* at sweep *i* was designed and implemented within the Metropolis equilibration phase; its mathematical definition is the following (Dai et al. 2007):


(8) $$S_{i}=f_{i}S_{i-1}$$

$$\begin{array}{r} f_{i}=1\;if(r_{\min}\leq r_{i}\leq r_{\max})|\left(S_{i-1}<S_{\min}\right)|(S_{i-1}>S_{\max}) \end{array}$$

$$=\;f_{i-1}\;if(r_{i-1}<r_{\min}\;\&\;r_{i}<r_{\min})|(r_{i-1}>r_{\max}\;\&\;r_{ni}>r_{\max})$$

(9) $$\;=\;\frac{1}{\sqrt{\;f_{i-1}}}\;if\left(r_{i-1}\left\langle r_{\min}\;\&\;r_{i}\right\rangle r_{\max}\right)|(r_{i-1}>r_{\max}\;\&\;r_{i}<r_{\min})$$

$$\;=\;\frac{2}{3}\;if(r_{\min}\leq r_{i-1}\leq r_{\max}\;\&\;r_{i}<r_{\min})(i=1\;\&\;(r_{1}<r_{\min})$$

$$\;=\;\frac{3}{2}if(r_{\min}\leq r_{i-1}\leq r_{\max}\;\&\;r_{i}>r_{\max})(i=1\;\&\;(r_{1}>r_{\max})$$

The values $r_{\min}$ and $r_{\max}$ are the low and high acceptance rate limits set by the user, and $r_{i}$ is the current acceptance rate. The constant $E_{S}$ sets the maximum and minimum step width when the stepwidth is in the target range:


(10) $$S_{\min}=E_{S}{\bar{S}}_{i}$$

(11) $$S_{\max}=\left(\frac{1}{E_{S}}\right)\;{\bar{S}}_{i}$$

(12) $$\bar{S_{ni}}=\frac{1}{i}\overset{i-1}{\underset{n^{\prime }=0}{\sum }}\;S_{n^{\prime }}$$

(13) $$E_{S}=\;10^{-4}\ldots 10^{-6}$$

**Fig. 1.**
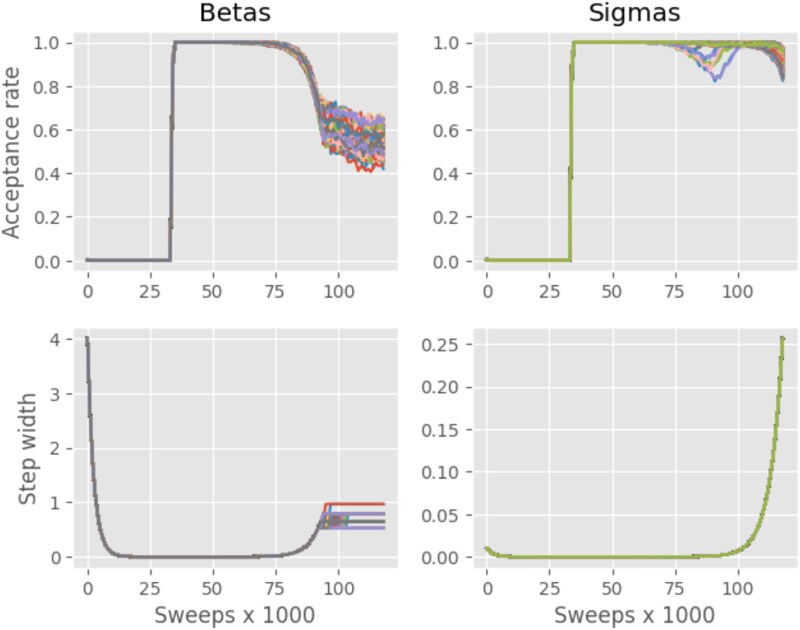
The acceptance rate and stepwidth for chromosomal region effects (βorBetas) and accession square root of the variance components ($\sigma_{i\;}$or sigmas) are plotted against sweep in a MCMC Experiment under the mixed linear model for dry weight using all available data. The acceptance rate was the fraction of accepted updates in a moving average of 1,000 sweeps. Stepwidth was defined as in Equation (9) and was updated every 1,000 sweeps.

Reasonable results were obtained with the stepwidth adjuster in our Metropolis algorithm implementation as shown in Fig. 1. The stepwidth adjustor takes the acceptance rate to the range 50 we desired during the equilibration phase. The step width adjuster stabilized for the chromosomal effects but increased for the variance components to achieve the desired acceptance rate range. Sometimes it was necessary to use an acceptance rate above 70% to achieve system equilibration. The dry weight target acceptance rate was set between 0.3 and 0.7 for the mixed linear model.

### MINE

The assumptions of MINE are separated into 2 categories, the main assumptions and ancillary assumptions, which are not critical but convenient for MINE implementation.

**Fig. 2.**
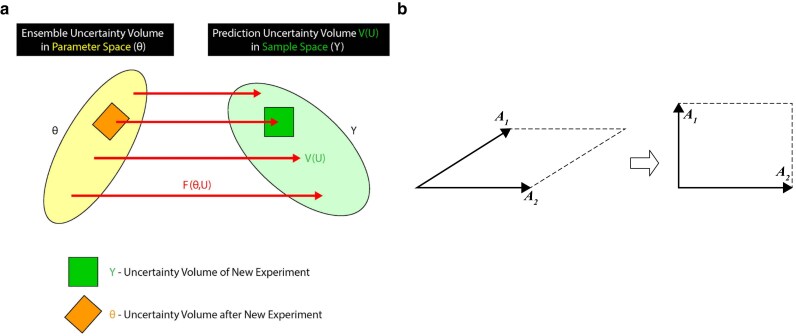
A visual explanation of the relation between the parameter space and phenotype space (Y). a) If we maximize the volume (green square) of our phenotypic observations on the quantitative trait, then the choice of parameters (brown square) will be shrunk. If we set up various experiments, adding 1 more experiment each time, then the next parameter choice will be better and volume, tighter. Panel (a) is derived from (Torres et al. 2025). b) If we randomly choose 2 models (A_1_ and A_2_) with different prediction vectors, they can be correlated under one experimental condition (LHS) or uncorrelated (RHS) or another experimental condition. Replace the vectors by their expectations. The completed parallelepiped or square has volume det(D) or det(E) depending on the MINE criterion chosen in (18) or (19).

1. There is a well specified collection of experiments in the future to choose from over time so that the modeling process is adaptive and evolutionary.
  MINE is an experimental design tool that is adaptive. It comes out of the traditions of solera in the sherry industry, iterative batch averaging methods for producing reference materials in Chemistry (Gouveia et al. 2021), response surfaces in Statistics (Box and Draper 1959), and quality control in industrial engineering (Eibl et al. 1992). Viewing the modeling as adaptive is also a key component to “computing life” in systems biology (Ideker et al. 2001; Dong et al. 2008) to discover underlying genetic networks from omics data.
2. There is an ensemble of models that are a reasonable description of the problem, such as for GWAS.
  The ensemble of models allows predictions about future experiments and helps guide the choice of experiments (Battogtokh et al. 2002; Landau and Binder 2021). The ensemble also summarizes past data accumulated on the problem. Both together allow the calculation of predictions of the systems in the future. The use of an ensemble addresses the situation in which the (number of data points *n*) << (the number of parameters *p*), as used in predicting the track of a hurricane. Mixed linear models are a standard modeling framework for GWAS (Zhang et al. 2010).
3. MINE is a discovery tool!
  The purpose of MINE is NOT to obtain a precise description of the model (Fisher 1935) but is there to use the models in the ensemble for discovery. The ensemble describes what is known and unknown about the system. As an example in GWAS, there are many possible SNPs that might contribute to a complex trait. The challenge is narrowing the field to those that matter through MINE. Once the field has been narrowed to the variables or relationships that matter, then classical design takes over (Fisher 1935).
4. As formulated, the moments of the sample statistics exist so that predictions about the future can be made.
  MINE works by using the ensemble to predict the behavior of sample statistics in the future (Dong et al. 2008). In order to carry out this prediction, it would be useful to have moments of these sample statistics exist. There are of course some systems where this assumption may not be valid with regard to the waiting time between solar flares and stock price changes as examples (Fama 1965; Akgiray and Booth 1988; Mandelbrot and Mandelbrot 1997; Leddon 2001; Lepreti et al. 2001). This assumption is one placed on the ensemble.
  There are also 3 ancillary assumptions:
5. There is a prediction uncertainty volume in the sample space of observations.
  In the MINE procedure it is necessary to have a measure of the volume in the sample space as in (18) and (19) and Fig. 2b. An ellipsoid is convenient, ad hoc, and tractable. Some other volume measure might be used. The choice of experiments is made to make this volume as large as possible to explore what is going on in the system. The secondary effect of increasing the prediction uncertainty volume is to reduce the uncertainty ensemble volume in the parameter space (Fig. 2) (Dong et al. 2008) through the model (Fig. 2a).
6. MINE can be conveniently applied if the process does not change over time (Fig. 6).
  This is an assumption which is worth checking and could be dealt with by adapting the model. In some situations, as this one in a GWAS, a farmer or agricultural scientist may not be interested in gene or chromosomal region effects that do not rise above the noise from season to season variation in the field. In our situation the environment acts as a filter for the genes that are truly significant that survive variations in rainfall and temperature with the growing seasons. When the model ensemble is descriptive of the process, it can be shown that MINE is consistent and converges to the true model as the sequence of experiments is made large in number (Dinh et al. 2014; McGee and Buzzard 2018), much as solera converges to a desired quality of sherry in practice.
7. There is a way to make an initial guess to the experiments to initiate MINE.
  Most large experiments have preceding experiments or pilot experiments, and these can be used to initialize MINE. If there are no such initial experiments, then the initial design X might be chosen to produce diverse outcomes. For example, in the case of a GWAS, the founding population used for the study might be chosen to be diverse genetically in the spirit of MINE to encourage exploration (Lasky et al. 2015) or as here, diverse in dry weight (Brenton et al. 2016) to lead to diversity in biomass in the current study. Now the description of MINE's application is presented in detail. The MINE approach, a model-guided discovery tool, is an experimental design tool to obtain the genotypes that yield the most trait information and its relation to chromosomal regions by maximizing the model prediction uncertainty volume about a complex trait (see reviews McGee and Buzzard 2018; Torres et al. 2025). This is done by 2 different criteria: covariance and Pearson correlation between predictions of the quantitative trait in the next GWAS experiment 1 yr later.

The covariance approach defines a volume (in the trait observation space) based on the uncertainty ellipsoid from the covariances of *K* observable predictions to reduce the volume in the parameter space (Fig. 2a). On the other hand, the correlation approach uses the Pearson correlation of *K* observable predictions for defining the ellipsoid. The MINE approach then uses the estimated parameters for the model by ensemble methods to calculate expected values of a predicted sample statistic *G_k_* in the next year.


(14) $$E_{mc}[G_{k}(.,X)]=N^{-1}\overset{N}{\underset{i=1}{\sum }}\;G_{k}(\beta^{i},X)$$

(15) $$E_{mc}[G_{k}(.,X)G_{j}(.,X)]=N^{-1}\overset{N}{\underset{i=1}{\sum }}\;G_{k}(\beta^{i},X)G_{j}(\beta^{i},X)$$

where *k* = 1,2,3 … *K*; *j* = 1,2,3 … *K*, and *i* = 1,2,3, …, *N*. In general, the dimension of the prediction vector need not be the same dimension as the number of genotypes to be selected in the MINE problem, but here it is formulated as such. The constant *K* also represents the number of genotypes to be chosen in a given year to obtain the maximum amount of information about the model parameters. As examples, the covariance and correlation criteria between predictions in the next experiment are based on a matrix of covariances or correlations, respectively, computed from (14) and (15). Each element of the covariance matrix is defined as follows:


(16) $$D_{kj}(X)=E_{mc}[G_{k}(.,X)G_{j}(.,X)]-E_{mc}[G_{k}(.,X)]E_{mc}[G_{j}(.,X)]$$

Each element of the correlation matrix can be obtained from the covariances:


(17) $$E_{kj}=\;\frac{D_{kj}(X)}{\sqrt{D_{kk}}\sqrt{D_{\;jj}}}$$

The matrix determinant represents the ellipsoid uncertainty volume, and since both matrices are square and positive semidefinite, then the determinant is computed in the following way from the eigenvalues of the covariance or correlation matrix:


(18) $$\det(D)=\;\overset{K}{\underset{k=1}{\prod }}\;\lambda_{k}$$

(19) $$\det(E)=\;\overset{K}{\underset{k=1}{\prod }}\;\chi_{k}$$

The *λ* and *χ* represent an eigenvalue from the covariance and correlation matrices, respectively calculated from the model predictions. These determinants also have the interpretation as a volume completed by the average covariance or correlation between 2 randomly chosen members of the ensemble (Fig. 2b).

### Suboptimal algorithms for computing MINE

The objective of this algorithm is to reduce the size of each subset to be analyzed; the size should be a number that divides the total number of accessions to be planted. In the case here, every subset was chosen to be of size 3 because 81 accessions were planted (81 is divisible by 3). When all subsets are analyzed, the top n are chosen until completing the number of accessions to plant. Its description is as follows:

**Algorithm 2.** (Suboptimal Algorithm)

*Set p_t_ number of elements in a tuple*
*Set p_a_ number of accessions to be selected*
*Generate all combinations of p_t_ elements from the accessions*
*Score all tuples*
*Sort the tuples based on the score*
*Select the top p_a_ individual accessions from the tuples*
***return*** *List of top p_a_ accessions*

As an example, identify all triples of accessions in the list of 343 accessions available in BAP. Compute the MINE score det(D) for each one. Sort from high to low. Pick 81 accessions at the top of the list.

### Monte Carlo

This MINE computation algorithm intends to select a subset of size equal to the number of accessions to plant, and the rest of the accessions go into a pool as candidates for later choices (but currently categorized as not for use). After many steps, 1 accession in the subset and 1 accession in the pool are swapped, and the MINE score is calculated to decide if the change is an improvement by the MINE criterion. The algorithm is described as follows:

**Algorithm 3.** (Monte Carlo algorithm)

*Set p_a_ number of accessions to be selected*
*Set m number of swapping steps*
*Generate an initial random sample of p_a_ accessions*
*for m times do*
*Swap one accession from the sample with one from the accessions pool*
*Score the sample*
*Accept or reject the change using Boltzmann probability and then score as a Hamiltonian*
***return*** *Sample of p_a_ accessions*

As an example, randomly pull 81 accessions from the pool of 343 accessions in BAP to be used. Score the 81 accessions for their det(D) score. Randomly swap with BAP pool m times using the Metropolis Algorithm 1.

### Suboptimal combination algorithm (Nc3+2)

This MINE computing algorithm combines 2 suboptimal search approaches; the length of subsets in both must be small. A suboptimal search is performed first. The top subset is taken, and from that point the search with the smaller subset length suboptimal search is performed after the top subset is taken until the total number of accessions to be planted is achieved. In our case, the first suboptimal search was length 3, and the second one, length 2. This algorithm is the following:

**Algorithm 4.** (Suboptimal combination algorithm)

*Set p_a_ number of accessions to be selected*
*Set p_t1_ number of elements per tuple in the first suboptimal algorithm*
*Set p_t2_ number of elements per tuple in the second suboptimal algorithm*
*Get the top tuple of length p_t1_ from the first suboptimal algorithm*
*Remove the accessions in the tuple from the pool*
*Create a list initializing the accessions in the tuple*
*while number of accessions in the list < p_a_ do*
*Run the second suboptimal algorithm for the accessions in the pool*
*Get the top tuple of length p_t2_*
*Add the top tuple to the accessions list*
*Remove the accessions in the tuple from the pool*
***return*** *List of p_a_ accessions*

As an example, form all triples from 343 BAP accessions. Score each by det(D). Get the top triple and remove all accessions in that triple from the BAP list. Add the top triple to the selected list for planting. Construct all doublets from the BAP list. Score each by det(D). Add the top doublet to the selected list and remove all accessions in that doublet from the BAP list. Go back to get another triple and doublet and cycle. Keep going until 81 accessions have been selected for planting.

### Greedy algorithm

This algorithm starts by taking as reference the top subset of the suboptimal search (Algorithm 2). It adds 1 accession at a time from the pool of other accessions, scores the subset, removes the previously added accession and goes on to the next one; after all accessions are passed, the highest score subset remains. The process is repeated until the remaining subset size equals to the number of accessions to be planted. Its description is the following:

**Algorithm 5.** (Greedy algorithm)

*.Set p_a_ number of accessions to be selected*
*Set p_t_ number of accessions per tuple in the suboptimal algorithm*
*Get the top tuple of length p_t_ from the suboptimal algorithm*
*Remove the accessions in the tuple from the pool*
*Create a list initializing the accessions in the tuple*
*while number of accessions in the list < p_a_ do*
*for each accession in the pool do*
*add the accession to the list*
*Score the list*
*Store the list and score*
*Remove the accession from the list*
*Sort the stored lists based on their score*
*Select the top list*
*Make the selected list the main list*
***return*** *List of p_a_ accessions*

Get the best triple from the 343 BAP accessions. Remove the 3 accessions from the BAP collection. Try adding 1 more accession from what remains in the BAP list, and add 1 accession for which the quartet formed has the highest det(D). The result is the best quartet. Remove the accessions in the quartet from BAP. Repeat the process to form a quintet until there are 81 accessions selected for planting, added one at a time.

**Fig. 3.**
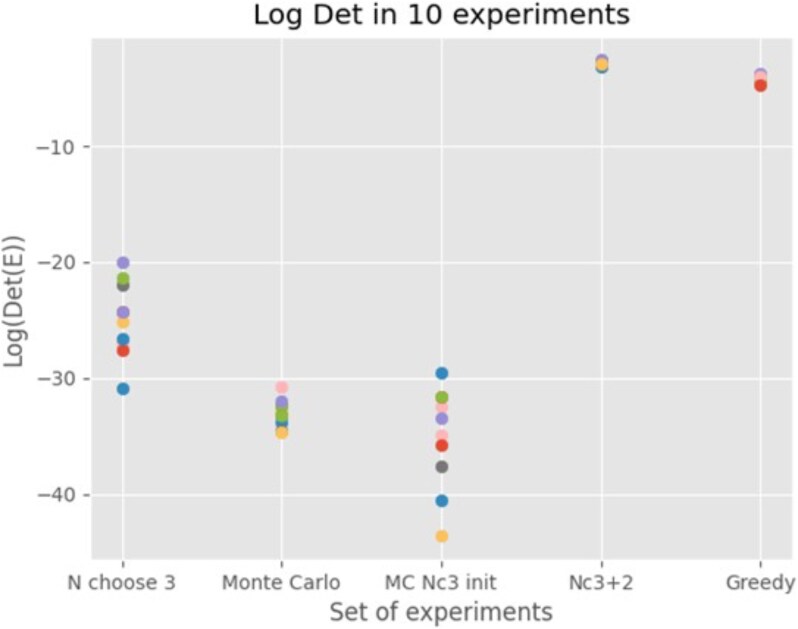
Among 5 distinct algorithms for computing the MINE experiment to select accessions for the next year, the best performing algorithms were “N choose 3” (Nc3 + 2) (Algorithm 4) and greedy algorithm (Algorithm 5) in 10 experiments. Each method was run on the same data but used a different random seed to repeat the experiment. Performance was measured by the log of the final MINE score in Equation (19).

To compare the performance of the 5 MINE computational algorithms over 10 different MCMC simulations were computed using the same data, but different random seed. The results were that the greedy and suboptimal combination algorithm was the best (Fig. 3).

### Marker selection

Most GWAS statistical analyses involve using t-tests, such as in Manhattan plots or quantile-quantile plots (Uffelmann et al. 2021). The limitation of this kind of approach is taking each chromosomal region or SNP one at a time in individual marker modeling; moreover, neighboring SNPs can be highly correlated. Since all markers here were encapsulated into a large design matrix and have only 1 model, a linear projection, Bayesian interval, and Benjamini–Hochberg criteria (Benjamini and Hochberg 1995) were used to filter out all but the most significant markers. The linear projection method is intended to remove parameters not constrained by the observations on the quantitative trait. The procedure is as follows:

**Algorithm 6.** (Linear projection algorithm)

*Create a block diagonal V*
*From design matrix X, create a matrix* $\;X^{T}V^{-1}X$
*Create a rotation matrix rot by extracting and sorting the eigenvectors from* $X^{T}V^{-1}X$
*Rotate the β vector to* $\;\beta^{*}=\;\beta rot^{T}$
*Remove the values that wandered around −∞ to +∞ that have* $\beta^{*}$ *near zero*
*Get the projected β by rotating back* $\beta^{*}$: $\beta^{p}=\beta^{*}rot$

The basic idea of Algorithm 6 is that the quadratic term in the Hamiltonian (2) is the one most sensitive to *β* as *β* components wander to plus or minus infinity. This wandering happens because some of the *β* components are not constrained by the data with *n* < *p*. To prevent this wandering in the leading quadratic term in the square error, $X^{T}V^{-1}X,$ its eigenvalues are computed and sorted into descending magnitude (along with the associated eigenvectors). The resulting eigenvalues from *n* + 1 on in the sorted list will be near zero and will have little effect on the quadratic term $X^{T}V^{-1}X$. Movement in the associated direction of the *n* + 1 eigenvector and beyond will be unconstrained in the random walk of the Metropolis Algorithm. A rotation ($rot^{T})$ to a coordinate system defined by the associated eigenvectors is carried out, and those $\beta^{*}$ coordinates are set to zero from *n* + 1 on to prevent a walk in these directions. Then, a rotation back (rot) to the original beta coordinate system is done to remove the wandering.

Once the linear projection method was applied, the Bayesian interval procedure was then applied. It consists of retaining those parameters outside of a 95% Bayesian confidence interval about zero. The Bayesian Confidence Interval is computed from the ensemble using Algorithm 7.

**Algorithm 7.** (Bayesian interval Algorithm)

*Sort each β parameter sample*
*for each β parameter sample do*
*if first 2.5% < 0 and last 2.5% > 0 then remove β parameter*

In parallel with the Bayesian interval Method (Jaynes and Kempthorne 1976), parameters were also filtered by the Benjamini–Hochberg criterion [9] using a false discovery rate threshold, in our case 0.05, and z-scores from the MC sample.

**Algorithm 8.** (Benjamini–Hochberg Algorithm)

*Get z-scores and p-values across all β parameters*
*Sort the p-values*
*Set a threshold α for false discovery rate*
*Set the Benjamini–Hochberg critical value* $BH=\frac{rank}{length\;of\;p\;vector}\alpha$ *BH = rank*
*if p-value < BH **then***
*Keep the β that corresponds to the p-value*

The markers that passed all filters were kept.

### Gene finder tool

**Fig. 4.**
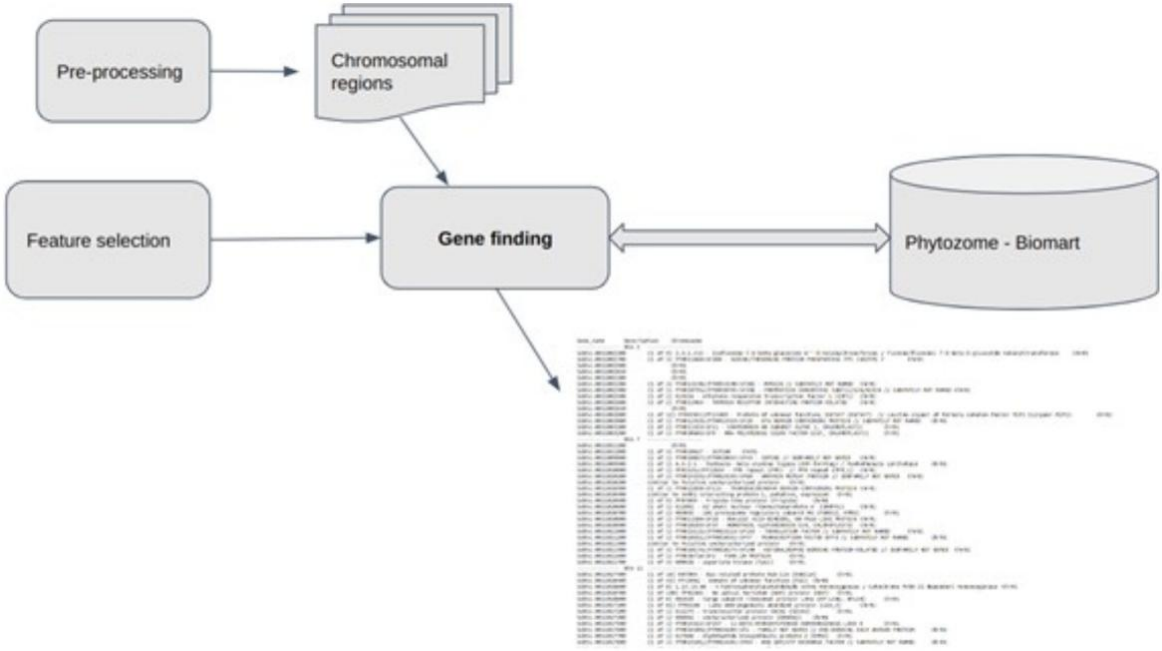
The gene finder program takes as input the preprocessing of a VCF file with the SNP data, the binning of the SNP data into chromosomal regions, and the chromosomal features selected as input and then uses Biomart (Goodstein et al. 2012) to retrieve the genes in chromosomal regions selected for output. The output includes the SOBIC number of each gene, its location, and some of its annotation.

Having the final chromosomal regions (markers) is only part of the results. A tool was implemented that allowed the retrieval of the known genes within each chromosomal region. This functionality can be expanded to sequences or other entities. This tool took the filtered chromosomal regions and its limits in the genome and connected to the Phytozome database via Biomart (Goodstein et al. 2012) (maintained by the DOE) to retrieve the genes within each region. The workflow for the gene finder is summarized (Fig. 4).

## Results

### Modeling

**Fig. 5.**
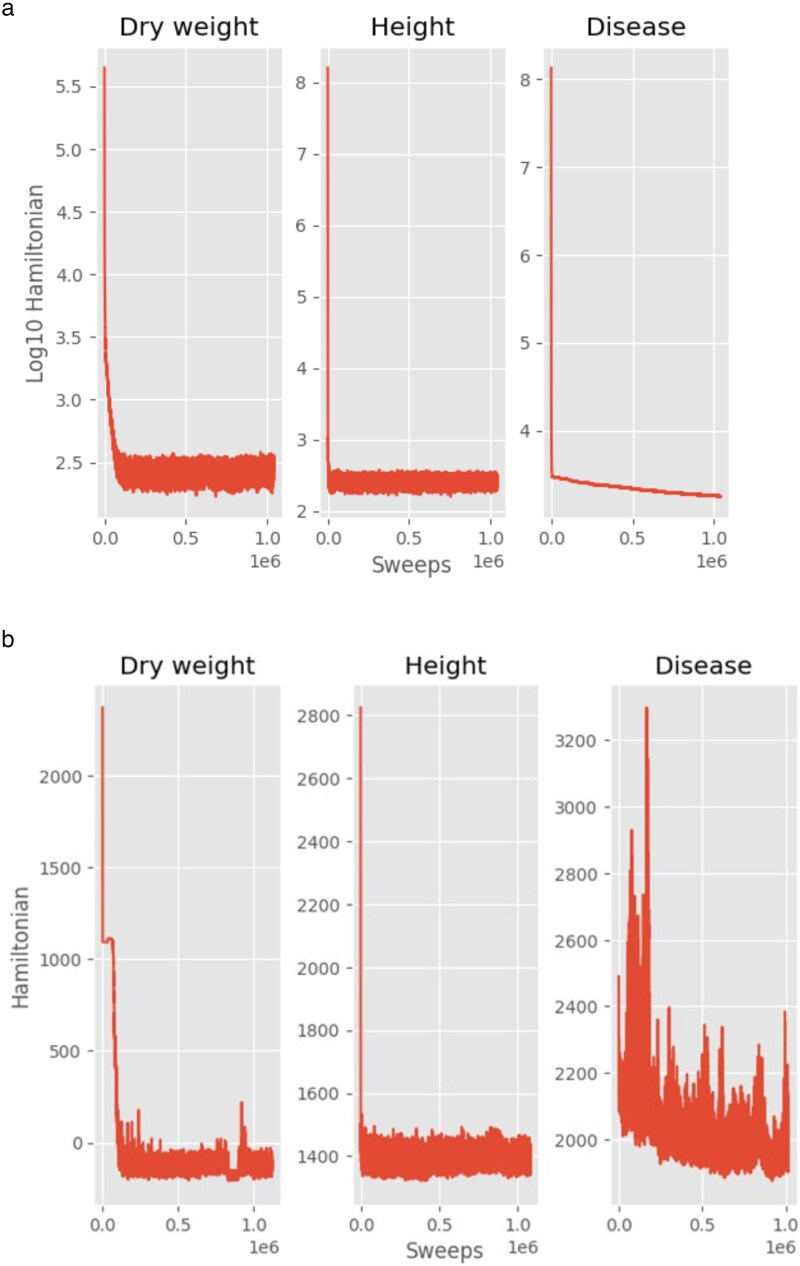
Dry weight, height, and disease Hamiltonian using Kresovich, year 1, year 2, and year 3 data against sweep (a visit on average to each model parameter once). a) Linear model; b) mixed linear model. The last term in panel (b). can take the Hamiltonian negative in some cases. The only key feature to the Hamiltonian is having a lower bound.

A 3 yr adaptive GWAS experiment using MINE was performed on Sorghum data. Our initial data (year 0) came from the Kresovich laboratory (Brenton et al. 2016), and from 3 consecutive years selecting different Sorghum genotypes (accessions) to be planted and harvested, creating an expanding database for dry weight, height, and disease. In year 1 our accessions were selected from the study made by Kresovich based on a diversity of dry weights. A total of 3 blocks of accessions were used for planting, and genotypes were planted in each block using a randomized block design with 3 blocks having 6–9 replicates per row. The parameter estimation procedure (computed with the Metropolis algorithm) performance was reasonable (Fig. 5), and a set of 1,000 sets of parameters were collected in the ensemble (MC sample) for each trait in the accumulation phase. The equilibration phase appeared successful (Fig. 5a) for the linear model and mixed linear model (Fig. 5b).

After each harvest on years 1–3, the dimensions of the Y vector and the number of rows of the design matrix X grew by the number of individuals added, including measurements on new BAP accessions. The number of columns of the X matrix remained fixed at 2,748 columns, the number of chromosomal bins spanning the *S. bicolor* genome. For year 3 and dry weight, the final number of rows (or individuals) was 2141. As new rows to the design matrix were added and integrated into the ensemble, the MINE procedure was carried out to select accessions for planting in the next year. The MINE procedure, based on the current ensemble with all the data up to the current time, guided discovery of relations between log dry weight and particular chromosomal bins.

In the second year the MINE procedure was used to select the genotypes to plant for year 3. A total of ∼81 genotypes were selected with MINE; 3 blocks were set up, each of them containing the genotypes randomly arranged into rows with 6 replicates. Data collected from the previous year were merged with the Kresovich data (year 0) to run the parameters estimation and the MINE procedure for year 3.

For the third year, data from years 2, 1, and Kresovich were combined to run the parameter estimation by the ensemble method, and the MINE procedure was computed for year 4. Again 3 blocks were used in the same location to plant the 81 new genotypes in year 3. In order to obtain the most significant chromosomal regions and their associated genes, a GWAS analysis was performed with the ensemble method using all of the data from Kresovich, year 1, year 2, and year 3.

As a control on the MC experiment, the Hamiltonians on both the linear model (Fig. 5a) and mixed linear model (Fig. 5b) were computed (a visit on average to each model parameter once) to demonstrate equilibration of the ensemble. The number of collected parameter vectors was 1,000 for the accumulation phase, and the number of decorrelation sweeps was 1,000 between each member of the ensemble in the accumulation phase. That is why Fig. 5a and b show the values of the Hamiltonian extending at least 1 million sweeps.

A variety of additional statistics were generated on our GitHub by the software including: (1) plots of parameters against sweep, stepsize against sweep, and acceptance rate against sweep to judge if the MCMC experiment was equilibrated. The reader may find it desirable to run the equilibration longer for disease.

**Fig. 6.**
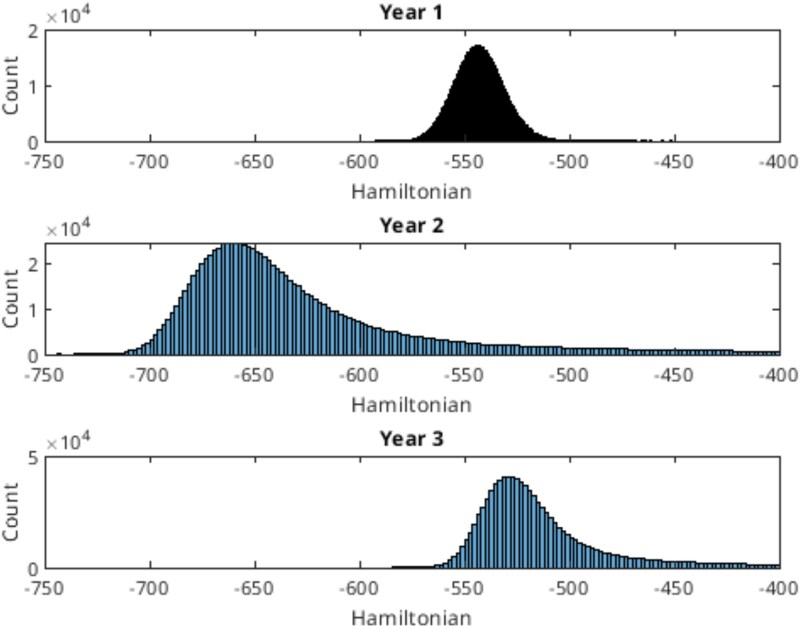
Ensembles separately fitted by year are overlapping with respect to their Hamiltonians (see GitHub). Hamiltonian histograms from ensembles of the mixed linear model for height were separately fitted in each year and computed.

An assumption of the MINE approach to GWAS (see next section on MINE) is that there is no year effect on the complex trait, and as can be seen in Fig. 6, the Hamiltonians for year 1, 2, and 3 are overlapping, suggesting no year effect. The Hamiltonian for a model with no year effects was compared with separate Hamiltonians for years (ie $H_{1}=-lnQ_{1})$ to calculate a likelihood ratio test (Λ): $-2ln\Lambda =\;-2(\ln Q_{\mathrm{no}\;\mathrm{year}\;\mathrm{effect}}-lnQ_{\mathrm{year}\;\mathrm{effect}})=-2(H_{\mathrm{no}\;\mathrm{year}}-H_{1}-H_{2}-H_{3})=35.0125\;\;\mathrm{on}\;\;2\;df\;\;\mathrm{with}\;\;P<0.0001\;$ for dry weight. The Hamiltonians chosen were the last ones in the MCMC run for each model. If the minima in the Hamiltonians are compared, then the likelihood ratio test statistic is −2lnΛ=−111.6007,whichfavorsthenullhypothesis. By Equation (3) the minimum in the Hamiltonian defines the maximum likelihood estimates (MLEs), and goodness of fit statistics like a likelihood ratio test or χ^2^ test have their asymptotic properties at the MLEs (Cramér 1999). In an MCMC run, there is no guarantee the local minimum found yields $Q_{\mathrm{no}\;\mathrm{year}\;\mathrm{effect}}\;\geq Q_{\mathrm{year}\;\mathrm{effect}}$. It is necessary to consider the distribution of Hamiltonians for each ensemble. There are 36 MCMC runs on the GitHub that allow all 12 fixed effects by block and year for dry weight, height, and disease.

To reconstruct the distribution of −2lnΛ, each of the Hamiltonians for the 3 ensembles with a year effect in Fig. 6 and the ensemble with no year effects were sampled 1,000 times. This produced a histogram for $-2ln\Lambda =-2(H_{\mathrm{no}\;\mathrm{year}}-H_{1}-H_{2}-H_{3}).$ The proportion (268/1000) of samples below zero are voting for the null hypothesis of no year effect. The proportion (732/1000) of samples above zero are voting for a year effect. These 2 proportions provide additional information by which to decide on a year effect.

When there is a year effect, 2 approaches are available. One approach is to introduce a year effect into the mixed linear model. Models are available that fit the year effects on GitHub. A second approach is to recognize that when there are small yearly effects, this environmental noise provides an additional filter for significant features in the genome. Some researchers are going to be particularly interested in chromosomal regions or SNP effects that rise above the yearly environmental effects of planting, such as those due to variation in rainfall.

### Maximally informative next experiment

The MINE procedure (with assumptions presented in the Materials and methods) was run to select the most informative genotypes for the second year. The data used were from Kresovich and year 1. The data for the third year was from Kresovich, year 1 and year 2.

**Fig. 7.**
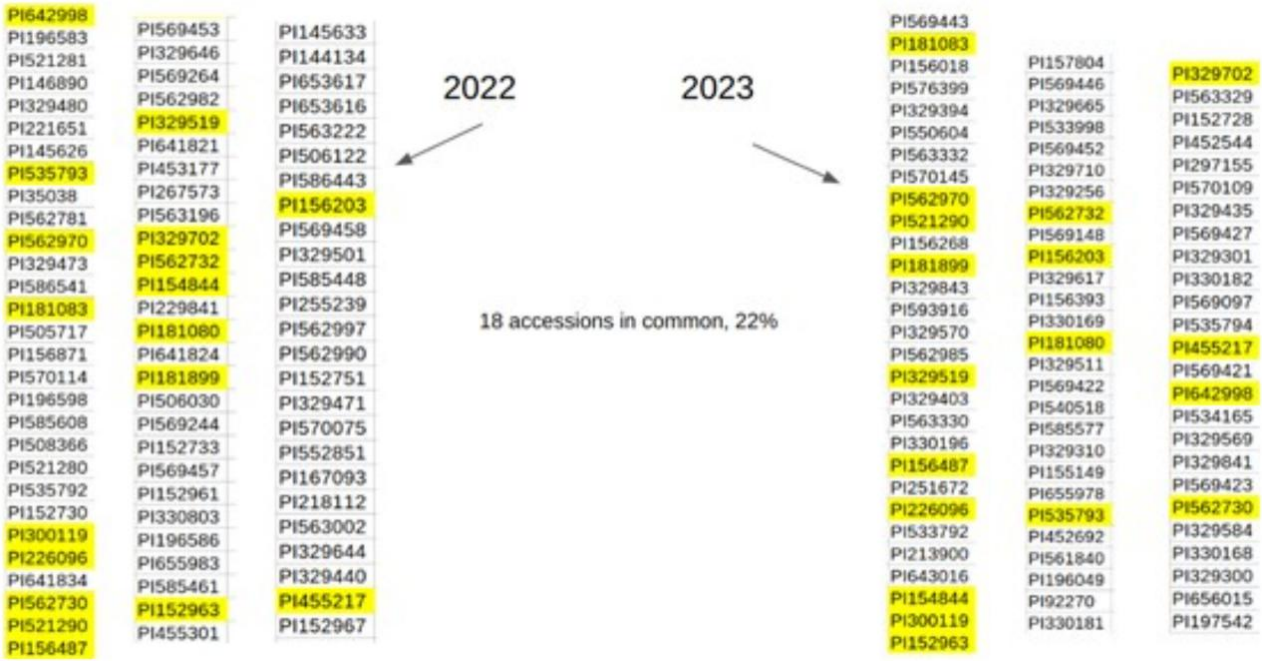
Accessions selected by the MINE procedure for planting in year 2 (2022) and year 3 (2023). The selected accessions are in yellow.

The MINE approach kept 22% of the accessions from year 2 to year 3 (Fig. 7).

**Fig. 8.**
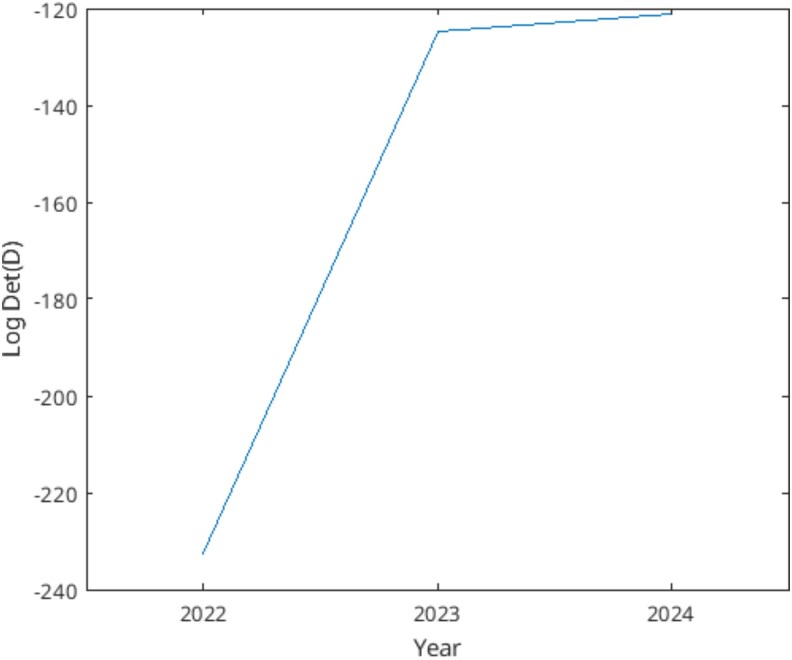
MINE score for covariance criterion det(E(D)) in Equation (18) increases over 3 yr. The MINE score was computed by the suboptimal Algorithm 2 in 2022 (being the only 1 available at that time) and by the Greedy Algorithm 5 in 2023 and 2024. Each prediction of log dry weight in the next year prior used all available log dry weight data in the ensemble of the previous year.

The MINE criterion (*D*) in (16), used to select the most informative accessions, increased in the experiment over years (Fig. 8), which shows that the MINE procedure was improving on our knowledge about the relation of a complex trait and its relation to the effects on the complex trait.

To understand the behavior of MINE a control was performed in year 1 in which accessions were chosen with a diversity of dry weights from a previous study (Brenton et al. 2016). It then seemed reasonable that any selection based on accessions with a diversity of dry weights should behave similarly to MINE. To test this idea, we tracked how MINE behaved in its choice of accessions over years 2 and 3 (Supplementary Table 1). It can be seen that the choice by diverse dry weights coincides remarkably well with the choice by MINE in year 3. For example, in year 3 MINE did go back to 54 accessions (which is 72% of the 75 accessions used in year 3) chosen on the basis of dry weight from (Brenton et al. 2016) in year 1 of our study. This coincidence in choices also explains why the improvement in MINE is diminished by the choices in year 1.

### Marker selection

**Fig. 9.**
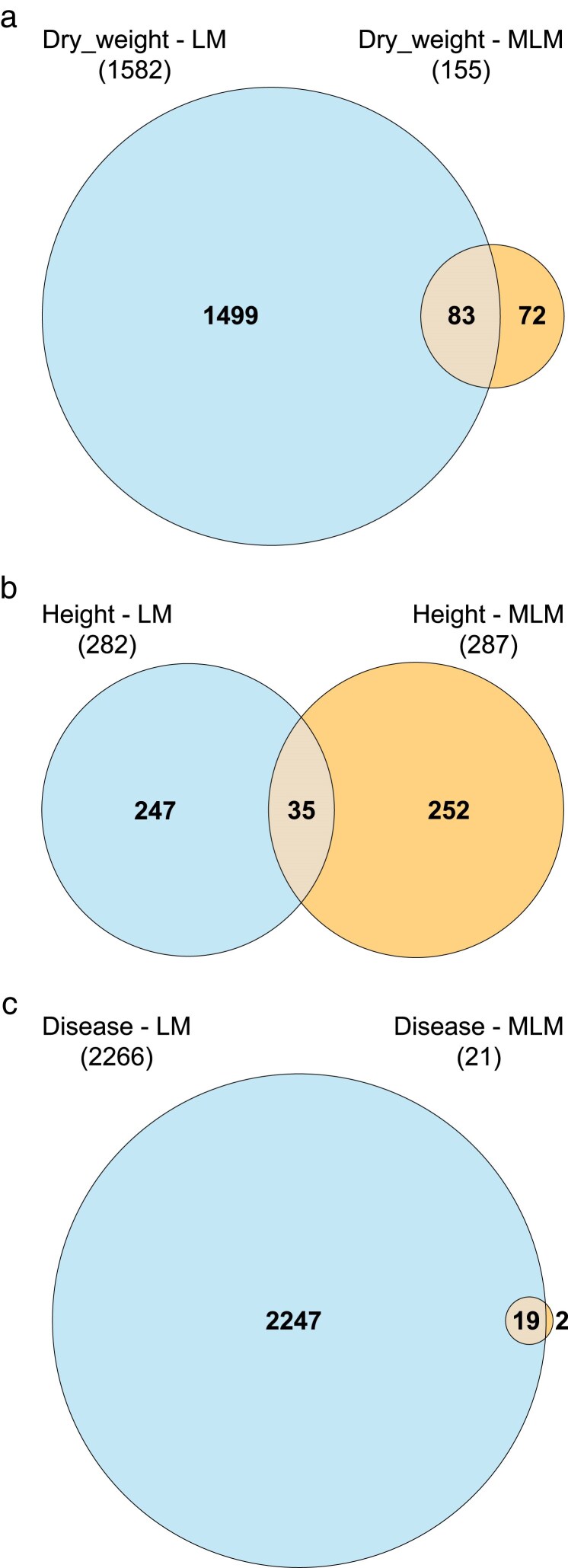
Significant markers (chromosomal regions) using all data available on disease selected simultaneously by the projection method (linear model with Algorithm 6), Bayesian Interval Method (Algorithm 7), and Benjamini–Hochberg method (Algorithm 8). LM are linear model results, and MLM mixed linear model results. Computed with (Heberle et al. 2015). a) Log dry weight. b) Height. c) Disease.

Markers were selected only using the final cumulative data in year 3, and both linear and mixed linear models were utilized in feature selection. The results of the feature selection of significant chromosomal regions with each class of models for each trait are summarized in Venn Diagrams (Heberle et al. 2015) (Fig. 9).

### Comparison of mixed linear model with fixed effects vs linear model in GWAS analysis

As seen in Fig. 9, the mixed linear model generally shows a higher filtering capacity for the effects of chromosomal region than the linear model. Only for height (Fig. 9b) was the filtering of features comparable for the linear and mixed linear models. This means our approach of attributing a variance component to each accession as a model parameter and also considering the fixed variance across observations translates into fewer selected features.

### Comparison of mixed linear model and linear model with a 1 at a time analysis

As a final comparison, the 2,748 chromosomal region effects were estimated “1 at a time” by regressing log dry weight Y using the final data set in year 3 on each column of the design matrix X separately, 2,748 times. The results are compared with those in a Venn Diagram with the 155 positive effects for chromosomal region found in Fig. 9a under the mixed linear model and 1,582 positive effects under the linear model with fixed effects. As with the results in Fig. 9a, the Benjamini–Hochberg feature selection method in Algorithm 8 was applied to the “1 at a time” approach. The results of a “1 at a time” strategy is 2,121 significant chromosomal regions, an overfit of positive effects, in comparison to 155 or 1,582 features selected by other methods in Fig. 9a. We conclude that the “1 at a time” method is not a reasonable strategy.

### Marker analysis

To understand what genes underly each complex trait, all of the genes within each selected chromosomal region was retrieved from the Phytozome database (Goodstein et al. 2012) using our “gene finding” tool previously described in Fig. 4. The markers from the mixed linear model were chosen because the filters showed a higher capacity as shown in the previous figures. The genes identified arose from an adaptive GWAS performed over 3 yr. Data were collected for 3 traits: dry weight, height, and disease; however, the construction of the design matrix selection (of genotypes) was guided by the dry weight observations. Then, an exhaustive search of the GWAS literature on each gene was carried out in sorghum, and all relevant sorghum GWAS papers were downloaded from Google Scholar. A script was created to take a gene found here in our GWAS and located in each paper. The results of this search are summarized in Supplementary Tables 1-3. The script was run on gene lists found in our GWAS for 3 traits. Disease had 241 genes in the GWAS here associated with significant chromosomal regions. Dry weight had 1,807 genes identified in 155 chromosomal regions, and height had 3,603 genes identified in 287 chromosomal regions. Due to the mating system only 1–2 genes as QTLs are needed to generate LD with the remaining 10–12 genes in a chromosomal region (Wang et al. 2013).

### Dry weight genes

Any gene listed in this section was found to have a significant effect on dry weight in our GWAS analysis (Supplementary Table 2). Its heritability score was 0.998996 and within the published range (Liedtke et al. 2020). Gene Sobic.001G112500 has been found to be important for biomass previously and is the closest gene to a significant marker, explaining 16.2% of the variation and making it the major one (Habyarimana et al. 2020). Gene Sobic.004G044200 and Sobic.004G273900 were related to tannin and starch content. Sobic.004G044200 was found 1,010 base pairs away from a selected marker, and Sobic.004G273900 was 33,720 base pairs (Kimani et al. 2020) from a marker. Gene Sobic.002G116000 was downregulated in mucilage-secreting aerial roots, and Sobic.010G120200 was determined to be a candidate by a previous GWAS and transcriptome analysis (Xu et al. 2023). Yield per panicle was related to gene Sobic.005G064900, which was found in LD with a significant SNP (Boyles et al. 2016). Gene Sobic.006G122200 was associated to biomass composition (Kumar et al. 2024). Tiller number was found to be associated with gene Sobic.007G151400 (and found here in its effect on dry weight) in a GWAS for forage yield (Wang et al. 2022), and gene Sobic.008G186400 in a GWAS for plant architecture and bioenergy (Luo et al. 2020).

Our GWAS approach on dry weight also recovered significant chromosomal regions containing genes from closely related traits such as height or disease, as supported by additional literature. So, genes below were pulled out in our GWAS as affecting dry weight, but in other GWAS studies affected other traits, such as disease or height. Amino acid traits affecting grain quality were related to genes Sobic.001G241200 and Sobic.001G405500, which are 21,770 and 4,080 base pairs away from an important marker (Kimani et al. 2020). Sobic.004G273600 was a direct hit for tannin content, and Sobic.004G273800 was 28,900 base pairs away from a significant marker (Kimani et al. 2020). Seed width was found to be related to gene Sobic.001G271500 (as well as dry weight here), its distance to a selected SNP being 22,206 base pairs (Ahn et al. 2023a); on the other hand, gene Sobic.007G093100 was related to seed perimeter and was located 37,632 base pairs away from a significant SNP (Ahn et al. 2023a). Gene Sobic.001G328500 was found near a significant QTL in a GWAS for grain color and tannin content (Zhang et al. 2023). For parasitic plant (Striga) resistance a previous GWAS found genes Sobic.002G021700, Sobic.009G056400, and Sobic.010G032000 that were close to significant markers (Kavuluko et al. 2021). Nodal root length was associated with gene Sobic.002G188800 in a previous GWAS as well as Sobic.002G188600 (Elias et al. 2024). Genes Sobic.002G280400, Sobic.002G280600, Sobic.002G280700, Sobic.002G280300, and Sobic.002G280800 showed resistance to anthracnose, downy mildew, grain mold, and heat smut (Ahn et al. 2023b). Sobic.002G416400 and Sobic.005G165632 were found to be a determinant for plant color (Wang et al. 2024). Leaf senescence was determined to be related to gene Sobic.003G052200 in a GWAS (Wang et al. 2023). Epicuticular wax genes were also discovered in our GWAS analysis. For example, Sobic.004G154200 was found 2,880 base pairs away from a significant marker, and Sobic.004G154300, 5,771 base pairs away from another SNP (Elango et al. 2020). A dwarf locus encodes a protein kinase, Sobic.006G067600, which was related to height (Enyew et al. 2022). Gene Sobic.006G067700 was found in 3 GWAS papers, and it was found in a dwarf locus (Bai et al. 2017; Miao et al. 2020; Kumar et al. 2024). An important marker representing 1% of the variance on days to flowering GWAS was found in gene Sobic.006G120000 (Enyew et al. 2022). A circadian rhythm gene was identified in a GWAS for forage yield Sobic.010G045100 (Wang et al. 2022).

### Disease genes

All genes listed in this section were identified as candidates in the GWAS analysis of Disease here and tied to previous GWAS results (Supplementary Table 3). Its heritability score was 0.648086 (Wenndt et al. 2023). For disease genes, we found Sobic.004G002200 related to starch content, which is an important factor in plant fitness under abiotic stress (Sapkota et al. 2020). Gene Sobic.004G002300 was associated with grain mold resistance in a GWAS performed by (Prom et al. 2020), locating it 0 base pairs away from a significant SNP. Resistance to parasitic plant (Striga) was also associated with gene Sobic.004G158901, where it was found in a significant association with a SNP (Kavuluko et al. 2021). A GWAS on seed morphology mentioned gene Sobic.004G340100 being 2,091 base pairs away from a selected marker, as well as gene Sobic.007G093100 being 37,632 base pairs away. Both genes were associated with the seed perimeter (Ahn et al. 2023a). Genes Sobic.006G149650 and Sobic.006G149700 were found associated with plant color (Wang et al. 2024).

### Height genes

All genes reported in this section were found to be associated with height in the GWAS analysis of this paper (Supplementary Table 4). Its heritability score was 0.273398 (Liedtke et al. 2020). Gene Sobic.003G202000 is associated with plant height and was found 40,100 base pairs away from a significant SNP (Luo et al. 2020). Gene Sobic.007G161700 was found close to a dwarf locus, specifically 2,000 base pairs away, and Sobic.007G161800 and Sobic.009G024600 were close to significant markers (Girma et al. 2019). Another gene found close to a dwarf locus is Sobic.007G160400, 94,100 base pairs away (Panelo et al. 2024). Height is also associated with gene Sobic.009G223500, which was found within 2 significant SNPs (Enyew et al. 2022).

Similar to dry weight, the height GWAS here also picked up significant chromosomal regions containing genes cited to be associated with other traits in previous GWAS literature, such as biomass or disease. Gene Sobic.001G270200 was related to grain mold resistance and was found 27,000 base pairs away from a significant SNP. Gene Sobic.001G270301 was also related to grain mold resistance and was found 166,000 base pairs away from another significant marker (Enyew et al. 2022). Anthracnose resistance was found related to gene Sobic.001G377200, which contains 2 significant markers (Cuevas et al. 2018). Gene Sobic.001G405500, Sobic.002G113600, Sobic.003G033900, Sobic.004G156000, Sobic.006G187900, and Sobic.010G080300 were found related to grain quality variation (Kimani et al. 2020). Gene Sobic.002G113900 was related to head smut resistance (Ahn et al. 2023b). Gene Sobic.002G208200 was related to grain yield, and gene Sobic.010G216600 was related to grain number and weight (Boyles et al. 2016). Tiller number was associated with gene Sobic.002G253000, which was found 3,700 base pairs away from a significant SNP (Luo et al. 2020). Genes Sobic.003G052200, Sobic.004G299500, Sobic.004G299600, Sobic.004G299700, and Sobic.006G261100 were associated with leaf senescence (Wang et al. 2023). Grain carotenoids were associated with gene Sobic.003G197500 and Sobic.007G156300, which were found 150,000 and 40,000 base pairs away from different significant SNPs (Cruet-Burgos et al. 2020). Response to anthracnose was related to gene Sobic.003G203500 (Ahn et al. 2021). Concentrations of iron and zinc were studied, and gene Sobic.003G350800 was reported highly expressed in a GWAS (Thakur et al. 2024). Epi-cuticular wax was associated with genes Sobic.004G154200, Sobic.004G154300, Sobic.004G156000, Sobic.005G222000, and Sobic.010G065300, which were found 2880, 5771, 3821, 7607, and 14,138 base pairs away from different significant SNPs, respectively (Elango et al. 2020). Gene Sobic.004G163700 and Sobic.007G004500 were found to be associated with parasitic plant striga resistance (Kavuluko et al. 2021). Grain color and tannin content were associated with gene Sobic.004G230000 (Zhang et al. 2023). Genes Sobic.005G033801 and Sobic.006G248300 were related to resistance to sorghum aphid and were found within 200,000 base pairs of different significant SNPs. Gene Sobic.010G091100 was found within 408,000 base pairs away from a significant SNP (Punnuri et al. 2022). Glume cover was found to be associated with genes Sobic.006G095550 and Sobic.006G095400 (Girma et al. 2019). Panicle exsertion was associated with gene Sobic.006G094600 and Sobic.006G094800 (Girma et al. 2019). Biomass accumulation under cold stress was related to gene Sobic.007G033300, which contained a significant SNP (Agnew et al. 2024). The number of nodes in aerial roots was associated with gene Sobic.007G155900, which contained a significant SNP (Wolf et al. 2024). Panicle length was found associated with gene Sobic.008G120200 (Wang et al. 2021). Anthracnose response was associated with gene Sobic.009G162500 (Ahn et al. 2022). Biomass related traits were associated with several genes, plant maturity, with Sobic.009G250500, Sobic.009G250600, and Sobic.009G250700; however, gene Sobic.009G250800 was the closest to a significant SNP, 55 base pairs away (Habyarimana et al. 2020). Biomass composition was found associated with gene Sobic.009G250600 (Kumar et al. 2024). Number of nodal roots had significant SNP found within gene Sobic.010G198000 in a GWAS for root system architecture (Elias et al. 2024).

**Fig. 10.**
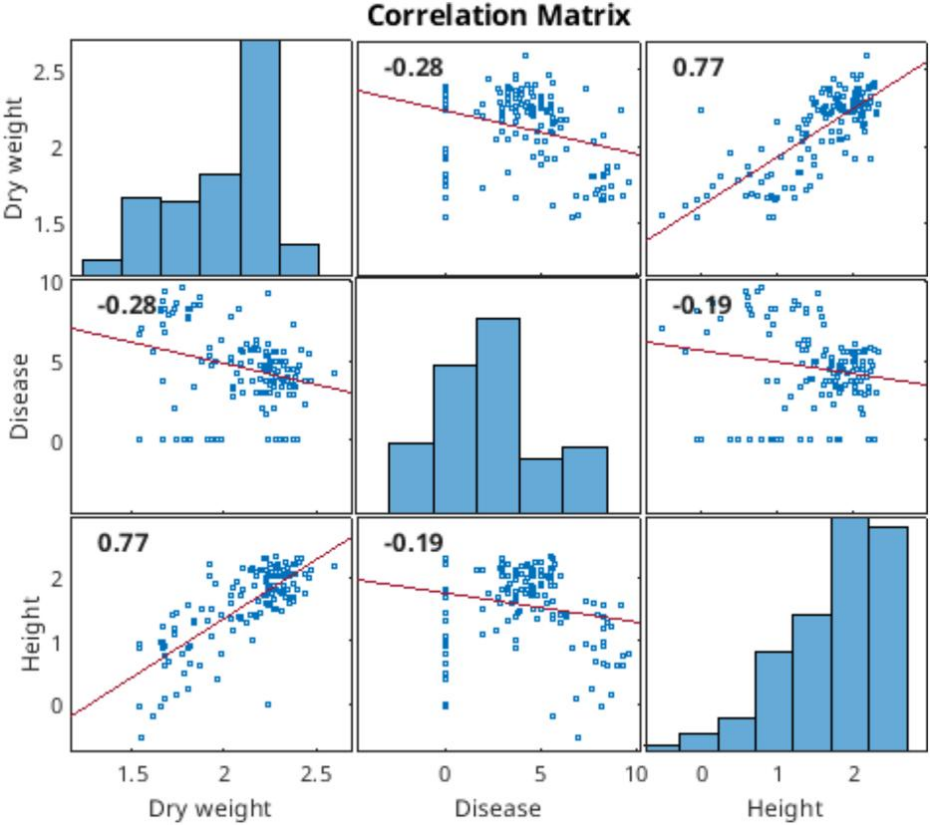
Dry weight, height, and disease phenotypic correlations and histograms based on data for all 3 yr.

In Fig. 10, there are phenotypic correlations displayed between dry weight, height, and disease; therefore, these correlations are consistent with some candidate genes appearing in the analysis of other traits in the previous section.

### Markers in the BAP original study vs our study

**Fig. 11.**
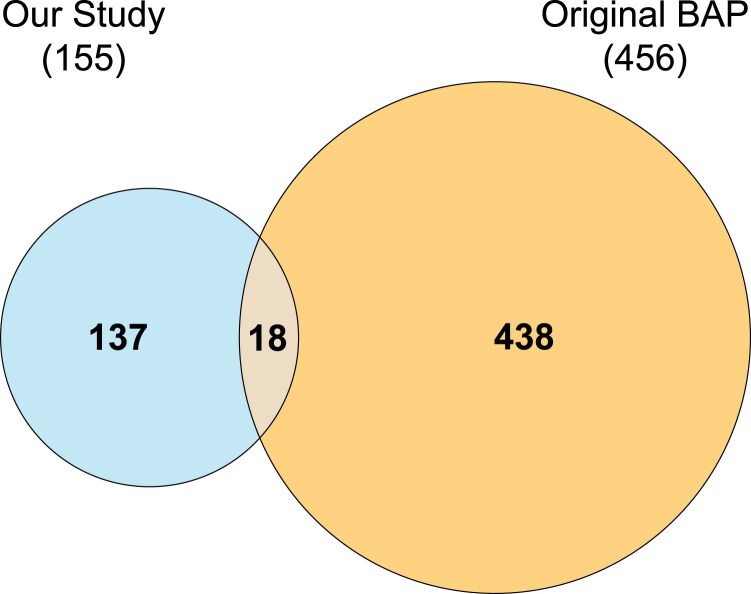
Selected chromosomal regions in the BAP original study (Brenton et al. 2016) and Arnold lab study for log dry weight under the mixed linear model. Computed with (Heberle et al. 2015).

The following Fig. 11 shows the number of selected chromosomal regions in the BAP original study and our study, using the same tools presented in this work as described in the Materials and Methods.

Brenton et al. (2016) implemented a large classical design on BAP that can be compared directly with the MINE experiment here. The original BAP study had 244 different accessions in its analysis (Brenton et al. 2016). The total number of accessions in the BAP collection is 343. 155 novel chromosomal regions were selected for follow up for experimental validation studies based on all of the 3 yr of data reported here. The MINE procedure took a subset of these 343 accessions each year; therefore, the MINE procedure ended up adding more individuals for some accessions and utilizing others in exploration, and that is what we see in the previous Venn diagram (Fig. 11). We confirmed the chromosomal regions for both our and BAP data alone against prior published GWAS studies in *S. bicolor*. There were 31 confirmed chromosomal regions contributing to dry weight in our study, resulting in a 20% contribution; on the other hand, there were 65 confirmed chromosomal regions contributing to dry weight in the BAP study, resulting in a 15% contribution. The ultimate validation of these chromosomal regions is further experiments examining the effects of genes through knockouts and expression studies within the chromosomal region.

To elucidate why the 2 studies in Fig. 11 had so little overlap a small simulation was conducted for height, one of the best studied traits in Fig. 9. Ten Y-vectors for height were generated using Equation (4) using the same X-matrix for all available height data in Fig. 9b under the mixed linear model. The 10 true *β* components used a range of values given in previous simulations in table 1 (Bouffier et al. 2014) and the variance components from Fig. 9b. That is, we setup height as complex trait controlled by 10 genes in 10 different chromosomal bins. The ensemble method in algorithm 1 was applied followed by 2 feature selection methods in Algorithms 7 and 8 for each of the 10 replicates in the simulation. The results were that about 1 in 10 of the true *β* components was recovered in the same ball park as the percentage of confirmations of previously reported studies by our results here. The false positive rate achieved was 0.08. The low power of both studies provides one possible explanation for the divergence in the Venn diagram in Fig. 11.

## Discussion

In this work we have presented a new GWAS experimental design approach supported by nontraditional methodology for adaptive and staged (in time) GWAS. Such sequential designs are not unheard of in Genetics (Sobel et al. 1986). The MINE procedure was proposed by Bernd Sch $\ddot{u}$ ttler( Dong et al. 2008) and has been previously used to identify the dynamics of cellular processes (McGee and Buzzard 2018) as well as to choose informative taxa in phylogenetic problems (Townsend and Lopez-Giraldez 2010; Townsend and Leuenberger 2011), both sets of prior work focusing on experimental design. Much like the phylogenetic problem in this work we have applied MINE to GWAS, in order to discover what genotypes are the most informative from an adaptive sequence of experiments over consecutive years for explaining a quantitative trait. The ensemble method including the MINE design tool were specifically designed for precision agriculture in which the number of effects (*p*) greatly exceeds the number of observations (*n*). Traditional approaches in experimental design cannot accommodate this situation (Fisher 1935; John 1998; Ryan and Morgan 2007). All of the methods in these summaries of experimental design consider only the case *n* > *p*. The result is that practitioners of experimental design use feature selection to fit the Procrustean bed of classical experimental design, as demonstrated in GWAS studies (Uffelmann et al. 2021). The second novelty of MINE is its focus on discovery rather than the precision of effects of genes or chromosomal regions on the complex trait of interest. In the situation where there are potentially 10^5^ effects but only 10^3^ observations (plants) in the field experiment, the focus must be on discovery of the important effects rather than the precision of the estimates of effects. The increase in precision through MINE is only a byproduct of the discovery process (see table 3 in Dong et al. 2008). The results here show us that MINE performs reasonably well (Fig. 8); there are limitations and benefits to using ensemble methods including MINE. The MINE design also outperforms the fixed classical design in the sense that MINE confirmed a higher proportion of previously reported chromosomal regions than the large classic design (Fig. 11).

While this GWAS does fall in the category of *n* < *p*, it does come close to being a classic design with 2,141 plants and 2,748 effects after 3 yr of data collection including the previous data (Brenton et al. 2016). In previous work, we examined classical power and type 1 errors under MINE with random rotation (Bouffier et al. 2014). To better understand how MINE here in practice was performing, previous simulations of MINE were considered under the simulated linear model with 10 QTLs to be discovered among 1,000 chromosomal bins (Bouffier et al. 2014). In these simulations, the experimenter had complete control of the design matrix X in contrast to the situation here where the experimental could only choose a row for the design matrix from 343 BAP accession genotypes. For the case with 1,000 plants observed in the sequence of simulated MINE experiments the false positive rate or type I error rate dropped below 0.3 at 1,000 plants in the simulated study, and the power to detect all 10 QTLs approached 1 (Bouffier et al. 2014). This is promising, but further simulations are in progress under the mixed linear model.

Embedding the model in the analysis of genetics experiments does require a change of viewpoint when Boltzmann's ensemble approach to accepting more than one model to describe the system is utilized (Guggenheim 1955). The ensemble is a collection of models summarized in a probability distribution over the parameter space with support from available data. MINE takes the ensemble approach a step further and allows the ensemble of models to evolve as MINE guides a discovery process (Dinh et al. 2014). As more data accumulate, the ensemble distribution tightens and refines our understanding of the true model as the uncertainty volume shrinks in the parameter space (Fig. 2). At each step in MINE, model averaging over the ensemble is performed to make predictions about system behavior and to guide future experiments.

One of the main benefits of ensemble methods including MINE is being able to perform an analysis having less observations than markers. Rather than utilizing a single SNP modeling procedure, all of the data are considered together. The main limitation is accommodating changes in the model over time. If the effects are stable in time, then smaller yearly experiments can be carried out to identify the effects and satisfy resource constraints (see Fig. 6). For example, our laboratory was only capable of planting 80 genotypes/accession per year, while still taking into account statistical design requirements, such as 3 randomized blocks containing 6 replicates of each genotype. The resulting field experiment still had 1,440 plants to manage, and only 720 (3 replicates per block) were harvested. Results show that around 20% of the genotypes were kept from 1 yr to the next one, achieving a good degree of diversity across the selected genotypes. The action of using MINE in year 3 and diverse dry weights to select accessions in year 1 had similar effects (Supplementary Table 4).

Finally, we filtered out the most significant chromosomal regions and examined all of the genes within each region; our literature review reveals that our GWAS was able to identify accurately markers containing genes related specifically to a particular trait in previous studies, such as height. Among the genes we found directly related to height, Sobic.007G161700 and Sobic.007G160400 were previously described by other GWAS studies to be associated with dwarfism, and genes Sobic.003G202000, Sobic.007G161800, Sobic.009G024600, and Sobic.009G223500 picked up in our GWAS were found directly influencing height. Genes found here and directly related to dry weight in other sorghum GWAS studies were Sobic.001G112500, Sobic.004G044200, Sobic.004G273900, Sobic.004G044200, Sobic.004G273900, Sobic.002G116000, Sobic.010G120200, Sobic.005G064900, Sobic.006G122200, Sobic.007G151400, and Sobic.008G186400. For disease, the genes directly related were Sobic.004G002200, Sobic.004G002300, and Sobic.004G158901 and confirmed in other GWAS studies.

We also showed that height, dry weight and disease traits have a degree of correlation (Fig. 10); therefore, our GWAS on height picked genes previously associated to dry weight and disease; similarly the GWAS on dry weight picked genes related to height and disease, and the same observation was made for the GWAS on disease. Interestingly dry weight genes found on height GWAS were Sobic.002G208200, related to grain yield, Sobic.010G216600, related to grain number and weight, and Sobic.007G033300, related to biomass accumulation under cold stress. There were many disease genes found in dry weight and height GWAS, the most interesting being Sobic.002G021700 Sobic.009G056400, and Sobic.010G032000, related to parasitic plant Striga resistance; Sobic.002G280400, Sobic.002G280600, Sobic.002G280700, Sobic.002G280300, and Sobic.002G280800, related to fungal resistance; Sobic.003G203500, related to response to Anthracnose; Sobic.005G033801, and Sobic.006G248300, related to resistance to sorghum aphid. Height genes found in dry weight GWAS were Sobic.006G067600, and Sobic.006G067700, related to dwarf loci.

In this work, we used 2 different models, a linear model with fixed effects and a mixed linear model. Both of them utilized the same chromosomal regions as explanatory variables; however, for the mixed linear model we added 2 terms to the Hamiltonian in (7) that allowed us to free the accession variances and convert them into additional parameters to represent genetic variance components. The result was a better filter for important chromosomal regions affecting the complex trait (refer to Venn diagrams in Fig. 9). The mixed linear model showed a higher filtering capacity, achieving a reduction of 90% in the final number of chromosomal regions for dry weight, and 99% for disease.

Several alternative approaches to computing a fit to mixed linear models were considered here. With respect to MCMC methods of ensemble identification, some other methods include Wang–Landau sampling (Landau and Binder 2021), particle swarm optimization methods (Caranica et al. 2020), parallel tempering (Landau and Binder 2021), and Gibbs sampling (Habier et al. 2011; Moser et al. 2015; Orliac et al. 2022). Wang–Landau sampling and Particle swarm optimization are useful when there are barriers between MCMC equilibria separated by mountains in the Hamiltonian. Parallel tempering is useful for a more careful exploration of the parameter space. Gibbs sampling is more likely to be trapped in local optima because a 100% acceptance of proposed moves in cyclical ascent of the ensemble through its margins. We chose not to pursue Gibbs sampling because of its cyclical ascent approach. We chose not to pursue Wang–Landau or particle swarm optimization because there was no initial evidence for a barrier problem (Caranica et al. 2020). We also chose not to use parallel tempering or particle swarm optimization because they build on the approach of Metropolis, but they are more exhaustive in their search strategy. We implemented then a standard baseline with Metropolis, which would allow us to judge whether or not more computational effort was needed to search the parameter space for good solutions during equilibration. All of the methods should involve the use of controls as in Figs. 1 and 5. Particle Swarm Optimization (Caranica et al. 2020) would be worth exploring in the future.

Another alternative to the sum method (Romagnoni et al. 2019) used here (see Materials and methods) to sidestep the LD problem is to incorporate LD into SNP feature identification on a whole genome scale (Habier et al. 2011; Moser et al. 2015; Orliac et al. 2022). Our approach to handling LD is 2 tiered. At level 1 a GWAS in the BAP panel is completed and described here; at level 2 a nested association panel (NAM) from BAP is underway for fine-scaling mapping of traits (Boatwright et al. 2021). The sum method has recently been vetted by a careful comparative study with other approaches (Romagnoni et al. 2019). It was also known that LD extended over 3.5–35.5 kb in the *S. bicolor* genome (Wang et al. 2013) because of its high selfing rate of 0.85. The mating system will generate high LD (Allard 1975). There is less interest here in LD due to the mating system within a chromosomal bin that contains about 10 genes each and more interest in associations due to linkage of SNPs to the complex trait of interest. Selecting a bin size of at least 50 kb would then sidestep issues of LD arising from the mating system and leave resolution within a bin to the NAM mapping population mapping experiments. As a final note on LD between a region and a putative QTL, about 20% of the promising regions are being retained, but the remaining 80% represent new explorations of the genome. MINE is not greedy. It is an exploration tool.

The use of MINE challenges the way experiments are done in precision agriculture on the home turf of the subject of experimental design (Fisher 1951). The focus of the MINE approach is on discovery of relationships in large data sets rather than the precision of effects in the experimental design. The parameter space is large and potentially could involve 105 effects when there are only on the order of 103 observations. This is a common problem in systems biology. The solution to this problem is not only using ensemble methods to address the *p* > *n* problem, but also having an adaptive approach that combines both intelligent data collection and the use of the model to guide future experiments. Model-guided discovery then leads to relationships with the underlying genes that can be tested in the context of classical experimental design, once the relationships between the complex trait and genes are found. The solution proposed here for GWAS is only one example of where ensemble methods and MINE are critical for the new omics experiments in genetics (Torres et al. 2025).

## Supplementary Material

jkaf163_Supplementary_Data

## Data Availability

Data and code are available at https://github.com/JArnoldLab/MINE along with a small working example for trial to insure FAIR principles are achieved in practice (Wilkinson et al. 2016). The VCF files from (Hu et al. 2019) are available at: https://doi.org/10.6084/m9.figshare.28548572.v1 Supplemental material available at G3 online.
